# Inhibition of HuR/ELAVL-1 attenuates fibrotic progression in Mdx mice with dilated cardiomyopathy

**DOI:** 10.1007/s00018-025-05979-0

**Published:** 2025-12-30

**Authors:** Andrea Farini, Monica Molinaro, Debora Mostosi, Mattia Camera, Michele Russo, Emma Leonetti, Mirella Meregalli, Lucia Prandi, Carla Liaci, Alessandra Ghigo, Emilio Hirsch, Giorgio Merlo, Yvan Torrente

**Affiliations:** 1 Neurology Unit, Fondazione IRCCS Ca’ Granda Ospedale Maggiore Policlinico, Milan, Italy; 2 Stem Cell Laboratory, Department of Pathophysiology and Transplantation, Dino Ferrari Centre, Università degli Studi di Milano, Neurology Unit, Fondazione IRCCS Cà Granda Ospedale Maggiore Policlinico di Milano, Via Francesco Sforza 35, Milan, 20122 Italy; 3 Molecular Biotechnology Center “Guido Tarone”, Department of Molecular Biotechnology and Health Sciences, University of Torino, Via Nizza 52, Torino, 10126 Italy; 4 Present Address: Department of Neurosciences, Rehabilitation, Ophthalmology, Genetics, and Maternal and Children’s Sciences (DINOGMI), University of Genoa, Genoa, Italy; 5 Present Address: Eli and Edythe Broad Center for Regenerative Medicine, University of Southern California, Los Angeles, USA

**Keywords:** Duchenne muscular dystrophy, Dilated cardiomyopathy, RNA binding proteins, HuR

## Abstract

Duchenne muscular dystrophy (DMD) arises from dystrophin deficiency, a crucial component of the dystrophin-glycoprotein complex (DGC) essential for maintaining cellular structural integrity by linking intracellular actin filaments to the basal lamina. Dysfunctions within this complex, coupled with increased inflammatory immune cell infiltration, contribute to the onset of dilated cardiomyopathy (DCM). This cardiac condition, characterized by necrosis and fibrosis, significantly impairs left ventricular function. Despite various treatment approaches, reliable effects on these pathogenic mechanisms remain elusive. RNA-binding proteins play pivotal roles in modulating pathways often dysregulated in cardiac pathology. Notably, HuR, which is upregulated in fibrotic cardiac regions and modulates innate immune system activation, emerges as a promising target. We investigated HuR expression in cardiac tissues of mdx murine model of DMD and assessed the impact of its inhibition with regards to DCM progression. Our findings reveal that HuR is indeed upregulated in mdx mice, and its inhibition leads to attenuation of cardiac fibrosis and improvement in heart function. These preclinical results underscore the potential of targeting HuR for therapeutic intervention to mitigate DCM-associated pathological changes, warranting further exploration for the development of effective treatments.

**Supplementary Information:**

The online version contains supplementary material available at 10.1007/s00018-025-05979-0.

## Introduction

Despite etiological differences, muscular dystrophies (MDs) are commonly affected by muscle degeneration, weakness, chronic inflammation. The onset of inflammation and subsequent immune system activation are distinctive features of MDs. Even if inflammation is a shared feature among these pathologies, differences exist in molecules and pathways involved and – intriguingly – in cell infiltrates, suggesting a specific pathogenic mechanism accounting for each form of MDs [1]. Indeed, the cardiac involvement is differentially relevant in distinct MDs; specifically severe cardiac symptoms are described in Duchenne muscular dystrophy (DMD) - whose patients exhibit early diastolic dysfunctions, arrhythmia, ventricular dilation, hypertrophy, and decreased fractional shortening [2] - while myotonic dystrophy (DM) is characterized by cardiac conduction disorders, interstitial fibrosis and hypertrophy of myocardiocytes [3]. Instead, patients affected by Facioscapulohumeral muscular dystrophy (FSHD) suffered only from minor cardiac abnormalities, as arrhythmic alterations [4, 5].

The asynchronous cycles of muscle fiber degeneration in DMD lead to the depletion of satellite cell reserves and the subsequent development of fibrosis. This condition is exacerbated by the infiltration of immune subpopulations into muscles. These events are tightly regulated by the release of Danger Associated Molecular Patterns (DAMPs), oxidative stress, and calcium influx, all of which depend on damaged muscle fibers. Cardiac manifestations in DMD patients are typically diagnosed later than those in skeletal muscles but are prevalent in these individuals by the age of 18, resulting in fatal cardiorespiratory failures [6]. These cardiac features have gained significance in the last decade due to improvements in medical management, increasing patient lifespan and overall prognosis. The inflammatory background of DMD cardiac muscles leads to the development of myocardial fibrosis, culminating in dilated cardiomyopathy (DCM) [7]. Similarly, in the cardiac tissue of DMD murine models, such as mdx mice, the absence of dystrophin and other components of the dystrophin-glycoprotein complex (DGC) compromises membrane integrity, particularly affecting the costameres — structures that transmit mechanical force from the sarcomere to the sarcolemma and the extracellular matrix (ECM) [8]. The disorganization of costameres observed in dystrophin-deficient conditions contributes to impaired membrane permeability [9], leading to up-regulation of calcium, nitric oxide (NO), and nitric oxide-cyclic GMP pathways. These alterations exacerbate mechanical stress-induced membrane ruptures and promote mitochondrial dysfunction [10]. Combined with impairments in myocardial KATP channels that severely affect ATP levels, these events contribute to cardiac necrosis and fibrosis characteristic of DCM patients [11].

Following these premises, it was attempted to modulate the activation and proliferation of myofibroblasts or microenvironmental signaling while others tried to alter RNA-dependent pro-fibrotic pathways [12, 13]: unfortunately, all these therapies were blunted by limits in target selectivity and cardiotoxicity [14]. Similarly, angiotensin-converting enzyme (ACE) inhibitors and β-adrenoceptor antagonists were used to mitigate inflammatory cues in dystrophic cardiac tissues [15, 16], however reliable results are no longer achieved due to important adverse effects, as the generation of pro-inflammatory mediators, severe hypotension and diastolic dysfunctions [17].

A new class of drugs has been proposed, based on the inhibition of RNA binding proteins (RBPs). In physiological conditions, these play a crucial role in stabilizing mRNA and regulating various biological events, including inflammation, fibrosis, angiogenesis, cell death, and cellular proliferation. Dysfunctions in these processes are common in cardiac pathology [18]. Studies by Cothani and colleagues revealed that the transition from human cardiac fibroblasts to pro-fibrotic and inflammatory myofibroblasts is mediated by a group of TGF-β dependent genes, regulated by RBPs [19]. In particular, Pumilio RNA binding family member 2 (PUM2) and Quaking (QKI) were identified as central hubs in the regulation of fibrogenic pathways in the hearts of DCM patients [19]. Other studies demonstrated the key role of the RBP ELAV Like RNA Binding Protein 1 (HuR) in regulating cardiomyocyte hypertrophy, possibly through the activation of p38-MAPK and the pro-hypertrophic transcription factor NFAT [20].

Specifically related to cardiac pathology, HuR and other RNA-binding proteins have been proposed as therapeutic targets for myocardial infarction due to their roles in regulating cellular responses to hypoxia, oxidative stress, inflammation, and fibrosis. Patil et al. recently demonstrated that HuR inhibition in TGF-β1-treated cardiac fibroblasts suppresses myofibroblast differentiation, proliferation, and fibrosis development, potentially via modulation of cyclins D1 and A2 [21]. Inhibition of HuR improved cardiac functions, leading to the regression of left ventricular remodeling and decreased fibrotic development. HuR was found to be significantly present in fibrotic cardiac regions, suggesting a functional relationship. In animal models, the inhibition of HuR showed positive modulation of left ventricular (LV) mass and LV posterior wall thickness, indicating an improvement in hypertrophic and fibrotic cues [22]. In particular, HuR modulates the activity of WNT1-inducible signaling pathway protein 1 (Wisp1/Ccn4), that in turn coordinates the remodelling of ECM together with connective tissue growth factor (CTGF) and nephroblastoma overexpressed (CCN) proteins [23]. This process, in conjunction with Wisp1 and TGF-β, participates in the activation of cardiac fibroblasts into myofibroblasts, ultimately leading to uncontrolled fibrotic development [24]. Inhibition of HuR in the cardiac fibroblasts of adult mice blocked the expression of Safe-Secreted Frizzled Related Protein 2 (SFRP2) expression, leading to down-regulation of myofibroblast differentiation driven by TGF-β [25]. In diabetic patients, cardiomyopathy is a common complication in patients preceding chronic inflammation and cardiac cell death, common in heart failure. Jeyabal et al. demonstrated that in hyperglycemic condition these pathological events were upregulated through a TNF-α/NLRP3/caspase-1/IL-1β pathway mediated by HuR and miR-9 [26]. HuR has the capacity to regulate the mRNA expression of pro-inflammatory cytokines, particularly interferon-stimulated genes, in response to innate immune system activation [27]. Similarly, considering that cardiac sodium channel levels are modulated in DCM [28], it was assessed that HuR regulates the expression of *sodium channel a-subunit (SCN5A)* by stabilizing the transcription factor *myocyte enhancer factor-2 C (MEF2C)* [29].

Different small molecules that inhibit HuR activity are now studied in pre-clinical trial [30] and efficaciously utilized to limit cancer development in mice [31]. Among these molecules, the MS-444 – formerly known as a myosin light chain kinase inhibitor – is the most employed inhibitor of HuR. Its mechanism of action centers on disrupting HuR homodimerization and preventing its cytoplasmic trafficking. This, in turn, inhibits the post-transcriptional stabilization of AU-rich element-containing mRNAs, which are involved in pathways such as inflammation, apoptosis, and fibrosis, leading to their suppression rather than direct transcriptional inhibition [32]. Accordingly, different works described the effects of MS-444 in glioblastoma cells [33], in the animal model of malignant peripheral nerve sheath tumor [34] and in other tumors depending on the over-expression of the Programmed death-ligand 1 (PD-L1) [35].

In this study, we demonstrated the upregulation of HuR in cardiac tissue samples obtained from mdx mice, as well as in human DMD cardiomyocytes. To gain deeper insights into the involvement of HuR in the pathogenesis of DCM associated with DMD, we conducted experiments to evaluate the effects of HuR inhibition on the affected cardiac tissues of mdx mice. In light of these converging lines of evidence and our experimental data, we propose that HuR’s contribution in DMD-related cardiac pathology manifests prominently in fibrotic processes but is not exclusive to fibrosis alone. HuR likely influences apoptosis, inflammation, and metabolic pathways - all of which contribute to the initiation and progression of fibrosis. Therefore, the multi-step inhibitory effects of MS-444 on HuR function may produce broad ameliorative outcomes in the mdx mouse model of DCM. This integrative view underscores the therapeutic potential of targeting HuR to modulate fibrosis, as well as related pathological pathways in DCM.

## Materials and methods

### Animal ethics statement and treatment

All the procedures performed on living animals are complied with Italian law (D.L.vo 116/92 and subsequent additions), approved by local ethics committees and are adherent to ARRIVE guidelines, for the full and transparent reporting of research involving animals. This work was authorized by the Ministry of Health and Local University of Milan Committee, authorization number 859/2017-PR (5247B.35, 10/07/2017). 9-month–old (9m) wild-type (C57Bl) and mdx male mice were provided by Charles River. Mice (*n* = 6 per group) received intraperitoneal (IP) injections of MS-444 (BE-34776; MedChemExpress; 10 mg/kg body weight) [36], dissolved in PBS/5% N-Methyl Pyrrolidine (NMP) (Sigma-Aldrich) or vehicle control, 2 times/week for 40 days. This route of administration has been shown to be safe and effective up to 50 mg MS-444/kg body weight and the drug is released slowly [37]. Furthermore, detection of MS-444 in tissues for pharmacokinetics studies is hard to obtain since the detection sensitivity is reached at tolerated doses. Radiolabeling of the compound is not straightforward as the compound is a natural product isolated from *Actinomyces sp.* [37] so that to verify the functional inhibition of HuR upon MS-444 in intestinal tissue, c-myc – a well-known HuR target expressed in epithelial cells that is fundamental in colorectal cancer development – was chosen as a biomarker [36]. The weights of the animals (and the standard deviation) at the sacrifice were the following: C57Bl mice: 30,31 g ± 3,52; mdx vehicle: 33,43 g ± 3,52; MS-444 treated mdx mice: 34,3 g ± 2,29.

### Cell culture and reagents

Fibroblasts were isolated from the heart of 9m mdx and C57Bl mice (*n* = 5 each) as detailed in [38]. Following the sacrifice of mice, hearts were exposed and rapidly perfused with ice-cold perfusion buffer; next, tissues were washed delicately with ice-cold perfusion buffer, then ventricula were isolated, resuspended in digestion buffer (50 ml) and minced with sterile scissors. The fragments were put into falcon tube with digestion buffer (2 ml/sample), incubated at 37 °C for 20 min and grinded several times with pipette. We repeated these steps until we obtained a valuable digestion of tissues and then we proceed with cycles of digestion and centrifugation to harvest the cells to be treated (2 × 10^5^ cells per heart). Then, the cells were resuspended into medium composed of 10 ml DMEM + 10% fetal bovine serum (FBS) and 1% penicillin–streptomycin and we isolated fibroblasts that adhere to 0.1% gelatin-coated 10-cm plate. Accordingly, cells were seeded in 12-well or 24-well microplates: cardiac fibroblasts derived from 9m C57Bl and mdx mice were cultured in DMEM medium supplemented with 15% FBS. Cells were seeded at subconfluent levels (< 50% confluence) in 96-well tissue culture plates and treated with 20µM and 50µM of MS-444 48 h at 37 °C. 5 mg MS-444 was reconstituted in 2.1718 mL of DMSO (50 mg/mL), sonicated to prepare the 10 mM stocking solution – as indicated in the manufacturer protocol – and added to DMEM + 15% FBS to reach the final working concentrations [39, 40].

Cell viability was monitored using the trypan blue assay and the MTT assay as described in [41]. Relative cell survival was calculated as percentage relative to DMSO vehicle-treated controls. For the trypan blue assay, cells were seeded in 12-well plates at a density of 2000 cells per well and cultured. Briefly, every 48 h cells were trypsinized, centrifugated at 1500 × *g* for 10 min, resuspended in DMEM + 15% FBS and added to trypan blue in 1:10 ratio. Viable cells were counted using a counting chamber. Each experiment was done a minimum of two times. Cells destined to the trypan blue assay that reached a 100% confluence were seeded in 6-well plates or Petri dishes. For the MTT assay, cells were seeded in 24-well plates at a density of 1000 cells per well. Briefly, thiazol blue tetrazolium bromide was reconstituted in RPMI without phenol red to have a final concentration of 5 mg/ml. This solution was then filtered with a 0.22 μm filter and a volume equal to 1/10 of medium volume was added to each well. Cells were incubated for 4 h and - after the incubation - the supernatant in each well was gently removed and 300 µl of DMSO were added to each well. The absorbance of samples was measured using a microplate reader (GloMax^®^ Discover, Promega, USA) at a wavelength of 560 nm. At each time point, two wells per experimental group were analyzed and counted three independent times.

### Histological analysis

Animals were sacrificed by cervical dislocation and cardiac muscles of 9 m mdx mice treated with MS-444 and vehicle dystrophic and control mice were collected for both histological and biochemical analyses. Hearts were fixed in PFA 4% (w/v) in PBS overnight (ON) at 4 °C, and dehydrated in ethanol 70% ON at 4 °C. Then, hearts were embedded in paraffin and sliced into 5 μm-thick transversal sections using a microtome.

To detect cardiac collagen deposition, paraffin-embedded slices were dewaxed, rehydrated, and stained with PicroSirus Red solution − 0.1% Sirius Red in saturated aqueous solution of picric acid (Fluka, Buchs, Switzerland) - for 1 h at room temperature. Slices were then washed for 2 s with acidified water (5 ml glacial acetic acid freshly added to 1 L of distilled water), dehydrated, and mounted. The area of red positive staining (collagen) was measured with ImageJ (NIH, Bethesda, Maryland). The average percentage of fibrosis to total area was calculated in 6–8 images per heart.

For CD18 immunohistochemistry, paraffin-embedded slices were dewaxed, rehydrated and washed 3 times in PBS. Antigen retrieval was performed by incubating slides in citrate buffer (10 mM Sodium citrate, 0.05% Tween 20, pH 6.0) for 20 min at 95°. Slices were then washed with PBS, incubated with blocking solution (5% goat serum, 2% Bovine Serum Albumin, 0.1% TritonX-100 in PBS) for 1 h at room temperature, and subsequently with anti-CD18 antibody (1:50, Integrin β2 (E9O7W), Cell Signaling Technologies, #72607) in 0.1% TritonX-100 in PBS at 4 °C/ON. Sections were incubated with biotinylated anti-rabbit secondary antibody (Vector Laboratories, BA-1000) in 0.1% TritonX-100 in PBS, then with avidin and biotinylated HRP (Vector Laboratories, PK-4005) following manufacturer instructions, and subsequently with DAB substrate (Vector Laboratories, SK-4100). Finally, sections were dehydrated and mounted: CD18 + cells were counted using ImageJ (NIH, Bethesda, Maryland).

For Ki-67 and HuR immunohistochemistry, deparaffinization and antigen retrieval were performed using the BenchMark Ultra system (Roche Diagnostics, #05342716001). Paraffin-embedded slices were dewaxed in EZ Prep Solution (Roche Diagnostics, #950 − 102) at 72 °C and processed for antigen retrieval using ULTRA Cell Conditioning Solution 2 (Roche Diagnostics, #950 − 223) at 91 °C for 68 min. Slices were then washed in PBS with 0.1% Tween-20 three times and permeabilized in 0,5% TritonX-100 in PBS for 10 min. Endogenous peroxidase activity was blocked with 3% hydrogen peroxide for 10 min, following incubation in blocking solution − 5% Bovine Serum Albumin (BSA) in PBS - for 1 h at room temperature, and subsequently incubation with anti-Ki-67 antibody (1:50, AB16667, Abcam) or anti-HuR antibody (1:50, AB200342, Abcam) in 5% BSA in PBS at 4 °C/ON. Sections were incubated with biotinylated anti-rabbit secondary antibody (Vector Laboratories, BA-1000) in PBS, then with avidin and biotinylated HRP (Vector Laboratories, PK-6100) following manufacturer instructions, and subsequently with DAB substrate (Vector Laboratories, SK-4100). Finally, sections were dehydrated, mounted, and quantified using ImageJ (NIH, Bethesda, Maryland).

### Isolation of cells from Mdx cardiac muscle

Isolation of myocytes and non-myocytes from mouse hearts was performed according to the protocol described by Farrugia [42]. Briefly, hearts were perfused with EDTA buffer, enzymatically and mechanically digested and the cardiac cell suspension was centrifugated. The centrifugation allowed the separation of the supernatant (primarily non-myocytes) from the pellet (primarily cardiomyocytes). The cardiomyocytes pellet was re-suspended in PBS supplemented with 5% horse serum (HS), following a second round of centrifugation. The supernatant was discharged, and the pellet was snap-frozen in liquid nitrogen. Non-myocytes cells were separated into CD45 + cells (identified as hematopoietic cells), CD45– CD31 + cells (endothelial cells) and CD45– CD31– cells (fibroblasts) by using LS columns (Miltenyi Biotec, Cat#130-042−401), following the manufacturer’s instructions. Briefly, to obtain CD45 + cells, non-myocyte cell suspension was centrifugated and the pellet was re-suspended and incubated in Wash Buffer (Miltenyi Biotec, Cat#130-091−221) containing FITC anti-mouse CD45 antibody (Miltenyi Biotec, Cat#130-110−796), following incubation in Wash Buffer containing anti-FITC MicroBeads (Miltenyi Biotec, Cat#120-000−293). After incubation, cell suspension was applied onto the LS column and both CD45 + and CD45– cells were collected. CD45– cells were centrifuged, and the pellet was re-suspended and incubated in Wash Buffer with FITC anti-mouse CD31 antibody (Miltenyi Biotec, Cat#130-123−675). Cell suspension was then incubated in Wash Buffer containing anti-FITC MicroBeads and applied onto the LS column. Both CD45– CD31 + and CD45– CD31– cells were collected. CD45+, CD45– CD31 + and CD45– CD31– cells were centrifuged, the supernatant discharged, the pellet snap-frozen in liquid nitrogen and subjected to RNA extraction and digital PCR experiments as described below.

### Nuclear/cytoplasm fractionation from murine fibroblasts

We employed the Subcellular Protein Fractionation Kit for Cultured Cells (Thermo Scientific, number 78840) to separate and prepare cytoplasmic, membrane, nuclear soluble, chromatin-bound and cytoskeletal protein extracts from cultured cells. In brief, cardiac fibroblasts derived from 9m C57Bl and mdx mice were harvested with trypsin-EDTA and then centrifuge at 500 × *g* for 5 min, washed with ice-cold PBS and then 1 × 10^6^ of cells were transferred to a 1.5mL microcentrifuge tube and pellet by centrifugation at 500 × *g* for 2–3 min. Once the supernatant was discarded, following the addition of different extraction buffers containing protease inhibitors to the cell pellet - Cytoplasmic Extraction Buffer (CEB), Membrane Extraction Buffer (MEB), Nuclear Extraction Buffer (NEB) - and centrifuge/vortex steps according to Manufacture’s protocol, we obtained the nuclear and membrane extracts for proteomic characterization.

### Western blot (WB) analysis

Cells were harvested from 9 m mdx and C57Bl hearts and from fractionation procedure described above while cardiac tissues were isolated from 3 m/14m/18m mdx and C57Bl mice and 9 m mdx mice treated with MS-444 and vehicle dystrophic and control mice: total protein concentration was assessed as previously described [43]. Samples were resolved on polyacrylamide gels (ranging from 6% to 15%) and transferred to nitrocellulose membranes (Bio-Rad Laboratories, CA) while the WB for fibroblasts were performed on 4%−15% Mini-PROTEAN™ TGX Stain-Free™ Protein Gels (15 well, 4–15% precast polyacrylamide gel) (Biorad, 4568086). The membranes were incubated ON with the primary antibodies against: H3 (1:600; Millipore, 05–928); GAPDH (0411) (1:600, sc-47724, Santa Cruz Biotechnology-SCB); vinculin (1:600, MA5-11690, Invitrogen); TLR4 (1:500, sc-293072, SCB); TOMM20 (1:500, AB186735, Abcam); HDAC1 (1:500, MA5-1807, Invitrogen); RELb (1:500, sc-48366, SCB); ATG7 (1:500, sab4200304, Sigma-Aldrich); DRP1 (1:500, AB184247, Abcam); P62 (1:500, P0067, Sigma-Aldrich); TNFα (1:500, e-ab-40015, Elabscience); IL-6 (1:500, sc-57315, SCB); phosphor-SMAD3 (1:500, e-ab-21–040, Elabscience); SMAD2 (1:500, e-ab-32916, Elabscience); P38 (1:500, E-AB-32460, Elabscience); HuR (1:500, ab136542, Abcam); TGF-β (1:500, e-ab-81441, Elabscience); vimentin (1:500, ab92547, Abcam); MMP-9 (1:500, sc393859, SCB); TIMP-1 (1:500, ab86482, Abcam); OXPHOS (1:500, MS604-300, Abcam); IL-10 (1:500, ab189392, Abcam); S-100β chain (C-3) (1:500, sc-393919, SCB); Cyclin D1 (1:500, sc-8396, SCB); Fibronectin (1:300, AB2413, Abcam); PKC-α (1:500, 1632376, Abcam); HuR (1:500, 11910-1-AP, Proteintech); HMGB1 (HAP46.5) (1:600, sc-56698, SCB); Bcl2 (1:500, MA5-11757, Invitrogen); FH (1:500, E-AB-15051, Elabscience); ACO2 (1:600, E-AB-16130, Elabscience); IDH2 (1:600, E-AB-11319, Elabscience); MDH2 (1:600, E-AB-16130, Elabscience); OGDH (1:600, HPA020347, Sigma); CS (1:600, SAB2701077, Sigma); GP-x1 (1:600, AB-22604, Abcam); FOXO-1 (1:500, sc-374427, SCB). Following incubation, the membranes were detected with peroxidase-conjugated secondary antibodies (Agilent Technologies, CA) and developed by ECL (Amersham Biosciences, United Kingdom). We used the Stain-Free blot image as total protein loading control to normalize the data obtained with Mini-PROTEAN™ TGX Stain-Free™ Protein Gels while we used vinculin as housekeeping for the other gels.

### Digital PCR experiments

Hearts were collected, frozen in liquid nitrogen and pulverized. Total RNA was extracted using TRIzol reagent (Invitrogen, Carlsbad, CA), and its concentration was determined with a NanoDrop™ 1100 (NanoDrop Technologies, Wilmington, DE, United States). cDNA was synthesized from 1000 ng of total RNA using a cDNA reverse transcription kit (Applied Biosystems, Foster City, CA). Relative mRNA level was analyzed by real-time PCR (ABI 7900HT FAST Real-Time PCR system, Applied Biosystems, Foster City, CA) with Taqman assays, using the Universal Probe Library system (Roche Applied Science, Penzberg, Germany). The primer sequences of the detected genes are shown in Table 1.

**Table 1:** Sequence of primers used in digital PCR experiments

Gene	Left primer (5’−3’)	Right primer (5’−3’)
SERPINH1	TTCAGCCCTTGCTTGCCTC	ACACTTTTACTCCGAAGTCGGT
FN1	TCCAGGACAACAGCATCAGTGTCA	CCACAGTGGGTTGCAAACCTTCAA
TGF-β	TGGAGCAACATGTGGAACTC	GTCAGCAGCCGGTTACCA
Col1a1	CATGTTCAGCTTTGTGGACCT	GCAGCTGACTTCAGGGATGT
Col3a1	TCCCCTGGAATCTGTGAATC	TGAGTCGAATTGGGGAGAAT
Ctgf	TGACCTGGAGGAAAACATTAAGA	AGCCCTGTATGTCTTCACACTG
MMP-2	TGACTGTGACCATGACCGGG	CAGGACTCTCACAAGGTCGG
MMP-9	GCCATTCACGTCGTCCTTAT	CGCTACCACCTCGAACTTTG
Tnbs4	CAGACAGAGATGGCATTGGAGAC	GGTTACTGACATCAGGACAGCTG
Prelp	CTCCTTCAACATCTCCAACTTGC	CTGGGTCCCGTTGATTTTCTCTA
FBN1	CCCTGCGAGATGTGTCCTGC	TGTGTCCAGCGGGGCATTTG
Postn	AAGCTGCGGCAAGACAAG	GGGCTGTGTCAGGAGATCTTT
HuR	TGTTTTCTCGGTTTGGGCGAA	GACCATTGAAACTGGTAATTGCC
WISP1	CAGCACCACTAGAGGAAACGA	CTGGGCACATATCTTACAGCATT
Gsto1	AATGCTGTTTCCCCTCACTG	GGGATGGCAGTGAAGACTGT
αSMA	GACGTACAACTGGTATTGTG	TCAGGATCTTCATGAGGTAG
TNF-α	CCACCACGCTCTTCTGTCTAC	AGGGTCTGGGCCATAGAACT
P38	GACCCTGATGATGAGCCTGT	CAGGTGCTCAGGACTCCATT
TLR4	GGCAACTTGGACCTGAGGAG	CATGGGCTCTCGGTCCATAG
IL6	CCAGCCAGTTGCCTTCTTG	AGTGCATCATCGCTGTTCATAC
HMGB1	CCATTGGTGATGTTGCAAAG	CTTTTTCGCTGCATCAGGTT
P21	GACAAGAGGCCCAGTACTTC	GCTTGGAGTGATAGAAATCTGTC
FOXO1	ACATTTCGTCCTCGAACCAGCTCA	ATTTCAGACAGACTGGGCAGCGTA

### Echocardiography

Transthoracic echocardiography was conducted on MS-444 and vehicle control-treated 9mmdx mice, 40 days post-treatment, utilizing a small animal high-resolution imaging system (VeVo2100, VisualSonics, Inc., Toronto, Canada), outfitted with a 22–55 MHz transducer (MicroScan Transducers, MS500D). Anaesthesia was induced through inhalation of isoflurane 2% and maintained by mask ventilation with isoflurane 1%. To perform the analysis in the best physiological conditions and reduce hemodynamic variability, mice were got in supine position at 37 °C and epilated. Echocardiographic parameters were measured as previously detailed [44]. Echocardiographic parameters were measured at the level of the papillary muscles in the parasternal short-axis view (M mode). LV fractional shortening was calculated as follows: FS = ((LVEDD - LVESD)/LVEDD) X 100, where LVFS indicates LV fractional shortening; LVEDD, LV end-diastolic diameter; and LVESD, LV end-systolic diameter. Diastolic parameters were measured with tissue Doppler and pulsed wave Doppler techniques in the apical long-axis view. From the pulsed wave Doppler spectral waveforms, we measured the peak early- and late-diastolic transmitral velocities (E and A waves) to obtain the E/A ratio and E-wave deceleration time. All measurements were averaged on 3 consecutive cardiac cycles per experiment, and cardiac function was assessed when heart rate was 400–450 bpm.

### Statistics

To allocate the animals to inhibit HuR expression by means MS-444, we used the randomization within blocks, so that animal handlers were blinded regarding the treatment that the mice received throughout all the experimental procedures. Mice that suffered from clinical complications following the injection of drugs - inflammation or infection of the peritoneal cavity; laceration of abdominal organs (liver and intestinal organs of the upper quadrants, the bladder on the lower midline and the cecum in the lower left abdominal quadrant); internal bleeding or infection; respiratory distress - were excluded from the experimental plan and eventually sacrificed. To determine significance when comparing multiple groups’ means, we used One-way ANOVA followed by Tukey’s multiple comparison test while Student’s t-test to compare two groups assuming equal variances. In case of non-parametric test, we performed the Kruskal-Wallis test. In any cases, the difference among groups was considered significant as follow: * at *p* < 0.05; ** at *p* < 0.01; *** at *p* < 0.001; **** at *p* < 0.0001.

### Data and materials availability

All the data of the manuscript are available in the main text or the supplementary materials.

## Results

**Fig. 1:**
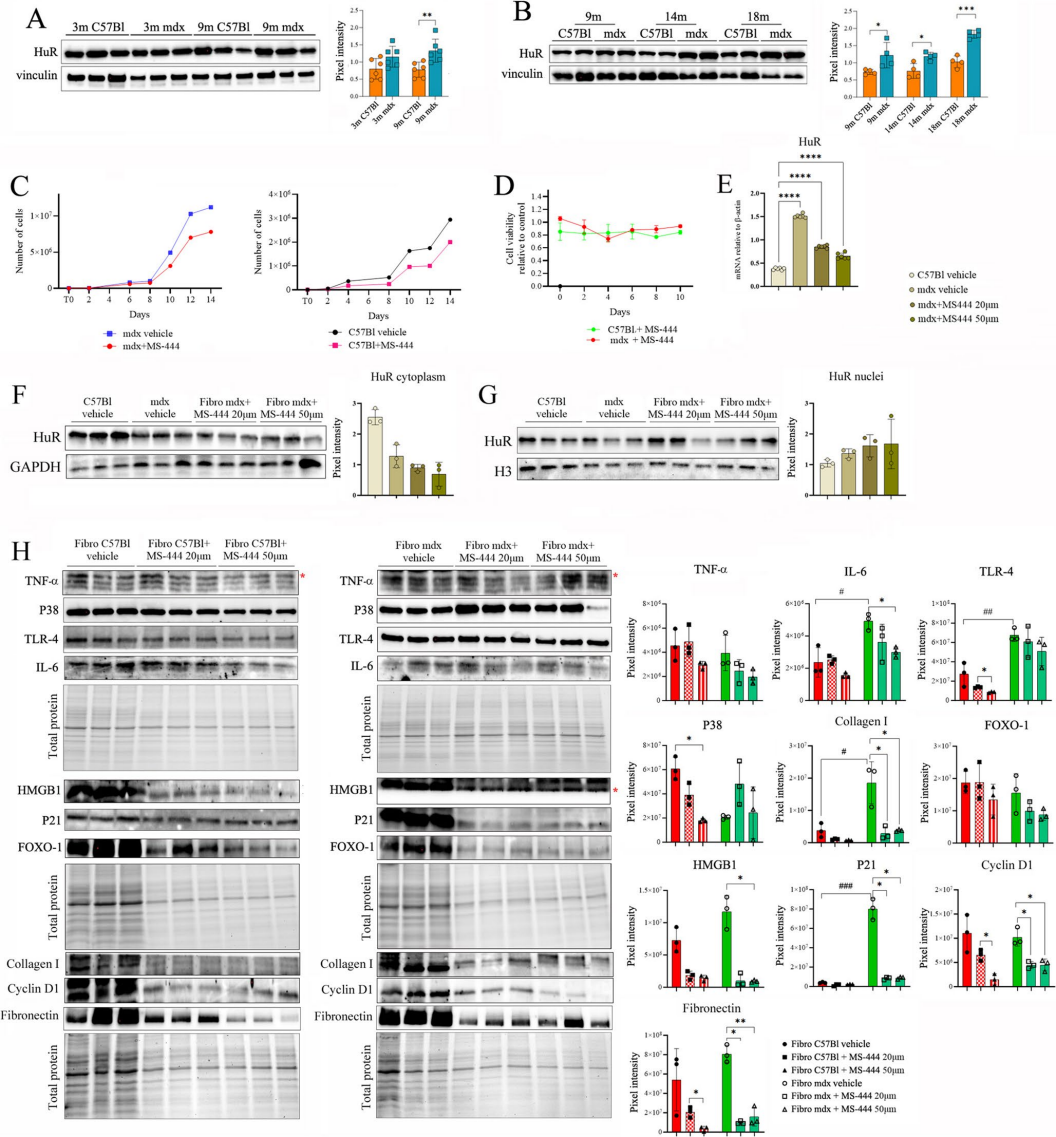
HuR expression in cardiac muscles of mdx mice of different ages and the effects of its modulation through MS-444 on cardiac fibroblasts. (**A**) Cropped images of representative WB analysis of cardiac muscle of 3 m mdx and 9 m mdx and age-matched C57Bl (*n* = 3 each, two independent experiments) mice showing the expression of HuR. (**B**) Cropped images of representative WB analysis showing the expression of HuR in cardiac muscles of 9 m, 14 m and 18 m mdx and age-matched C57Bl (*n* = 2 each, two independent experiments). (**C**) Proliferation and (**D**) cell viability (MTT) of cardiac fibroblasts harvested from mdx and C57Bl mice. (**E**) Digital PCR evaluation of HuR expression in cardiac fibroblasts treated with different concentrations of MS-444. Cropped images of representative WB analysis of HuR expression in cytoplasmic (**F**) and nuclear (**G**) compartments from cardiac fibroblasts treated in vitro. Normalization was carried out using histone H3 as a nuclear marker and GAPDH for the cytoplasmic fraction, confirming the specificity and efficiency of the fractionation. (**H**) Cropped images of representative WB analysis of cardiac fibroblasts treated with different concentrations of MS-444 to assess the expression of HuR, IL6, TLR4, P38, TNF-α; HMGB1, P21, FOXO-1; collagen I, Cyclin D1, fibronectin according to total proteins loading. Data information: densitometric data were normalized on vinculin and expressed as mean ± SD for WB. Stain-free gels were used for total protein quantification and expressed as mean ± SD. (*p < 0.05, **p < 0.01, ***p < 0.001 with Brown-Forsythe and Welch ANOVA tests). ^#^*p* < 0.05 and ^###^*p* < 0.001 with unpaired t test with Welch’s correction

To investigate potential associations between DMD-linked DCM and HuR modulation, we analyzed cardiac tissues from dystrophic mdx mice at various stages: pre-onset (3 m), onset (9 m), and during DCM progression (14m and 18m), comparing them to age-matched healthy C57Bl (WT) mice (Fig. 1). At the pre-onset stage, no significant difference in HuR expression was observed (Fig. 1A). However, as aging and DCM progression occurred, HuR expression continued to increase only in mdx mice (Fig. 1B).

To better define the potential impact of HuR-dependent mechanisms in dystrophic cardiac tissue, we investigated HuR expression across different cardiac cell types, with a focus on its possible role in modulating inflammatory pathways and innate immune system activity. Cardiomyocytes and non-myocyte populations—including hematopoietic CD45⁺ cells, CD45⁻CD31⁺ endothelial cells, and CD45⁻CD31⁻ fibroblasts—were isolated from the hearts of 9m mdx and C57Bl/6 mice (*n* = 3 per group), and HuR mRNA levels were analyzed via digital PCR (Supplementary Fig. S1). We observed a general upregulation of HuR expression in dystrophic cells compared to controls. Notably, HuR expression was most pronounced in fibroblasts, followed by CD45⁺ immune cells (Supplementary Fig. S1). Fibroblast proliferation and conversion to a myofibroblast state is a critical cellular process driving fibrosis and, therefore, a significant factor contributing to DCM [45]; this process was inhibited by targeting HuR in adult mice cardiac fibroblasts [25]. To dissect the effects stemming from cardiac fibrosis from those associated with HuR activity per se, cardiac fibroblasts were isolated from mdx and healthy C57Bl hearts for primary culture and treated with HuR inhibitor (MS-444, 50 µM every 48 h). Cell viability and proliferation were then quantified. These assays revealed that MS-444 slightly affected proliferation in cardiac fibroblasts from mdx mice (Fig. 1C, D), with a corresponding reduction in HuR expression in these cells (Fig. 1E). Then, we performed subcellular fractionation experiments of nuclear and cytoplasmic compartments from cardiac fibroblasts treated in vitro. The Western blots show a clear reduction of HuR protein in the cytoplasm (Fig. 1F) with a corresponding accumulation in the nuclear fraction upon MS-444 treatment (Fig. 1G). Normalization was carried out using histone H3 as a nuclear marker and GAPDH for the cytoplasmic fraction, confirming the specificity and efficiency of the fractionation.

Transcriptomic and proteomic analysis demonstrated that levels of pro-fibrotic and -inflammatory cytokines IL6, TLR4, and TNF-α were downregulated in MS-444-treated fibroblasts from mdx hearts compared to untreated dystrophic ones (Fig. 1H and Supplementary Fig. 2). Similarly, MS-444 determined a down-regulation of HMGB1-p21 axis of senescence [46, 47] (Fig. 1H and Supplementary Fig. 2). Among fibrotic response markers [48], FOXO-1 expression was affected by MS-444 treatment in cardiac fibroblasts from mdx mice (Fig. 1H and Supplementary Fig. 2). Further proteomic analysis showed a down-regulation of ECM components including fibronectin and collagen type I in treated cells (Fig. 1H). This is consistent with previous studies describing the role of HuR and HuR-regulated proteins in the fibroblast-to-myofibroblast transition in cardiac tissue [21, 24] as well as in the progression of pathological remodelling [49]. Intriguingly, collagen I was over-expressed in DMD fibroblasts related to CTR ones. Considering the known involvement of HuR in cyclin regulation [21, 50], we also demonstrated that MS-444 significantly modulated Cyclin-D1 in dystrophic cells and – at higher concentration – also in CTR tissues (Fig. 1H). Taken together, these data strengthen our mechanistic interpretation that MS-444 inhibits HuR function in cardiac fibroblasts predominantly by blocking its nucleocytoplasmic shuttling and RNA-stabilizing activity.

### Treatment with MS-444 in Mdx mice led to an improvement in cardiomyopathic features

**Fig. 2:**
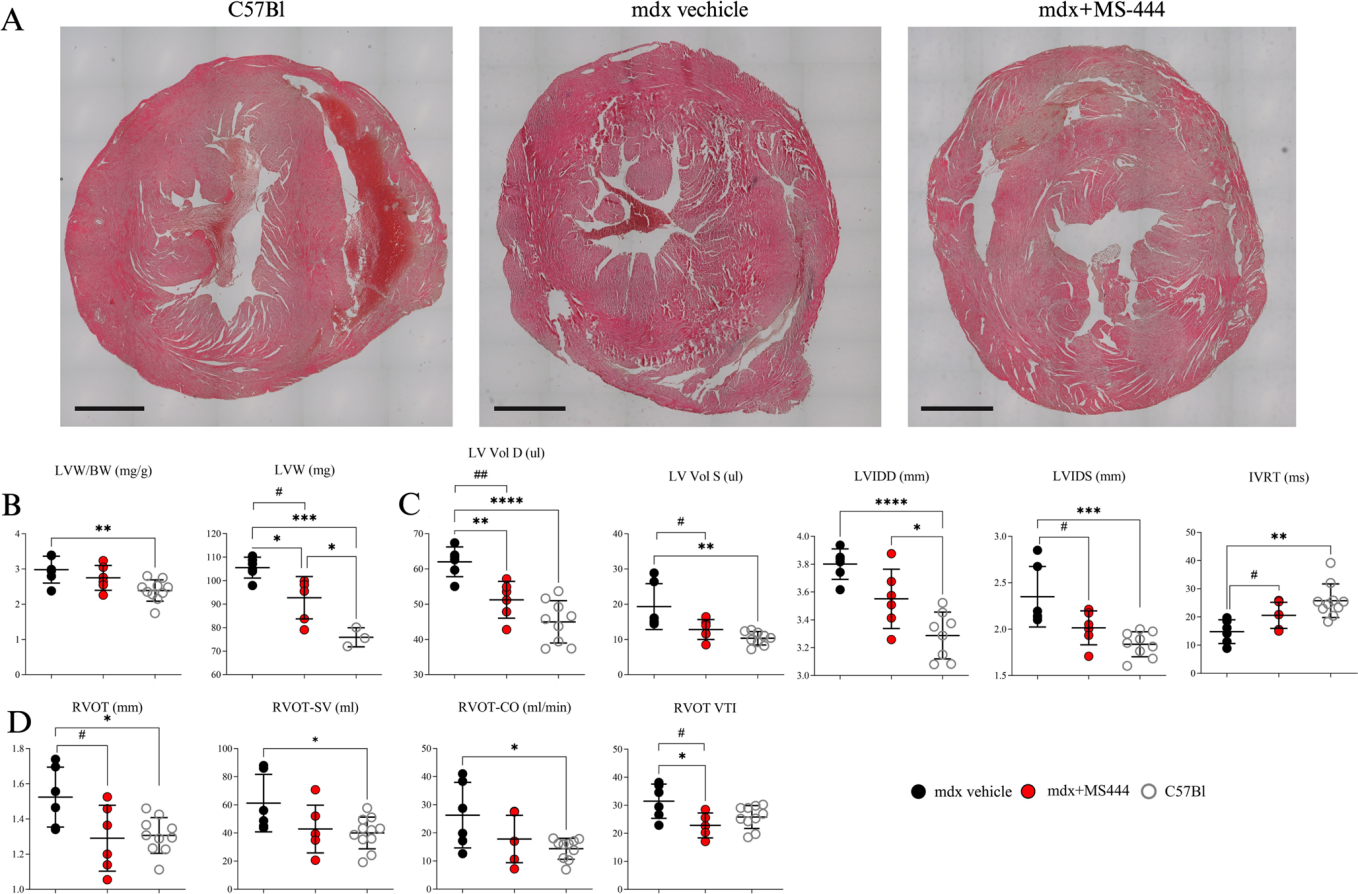
Left and right ventricular function in mdx mice following MS-444 treatment. Representative whole-heart images (scale bar: 1 mm) (**A**) and transthoracic echocardiography analysis in 9 m mdx mice treated with MS-444 and vehicle control (*n* = 6 each) and age-matched C57Bl ones (*n* = 11) (**B-D**). Abbreviations: LV volume in diastole (LV vol D); LV volume in systole (LV vol S); left ventricular weight (LVW) mass; LVW normalized to body mass weight (LVW/BW); Left Ventricle Internal Diameter at diastole (LVIDD); Left Ventricle Internal Diameter at systole (LVIDS); isovolumic relaxation time (IVRT); Right ventricular outflow tract (RVOT); Right ventricular outflow tract – stroke volume (RVOT-SV); Right ventricular outflow tract – cardiac output (RVOT-CO); RVOT-Velocity Time Integral (RVOT-VTI). Data information: *p < 0.05, **p < 0.01, ***p < 0.001, ****p < 0.0001 with Brown-Forsythe and Welch ANOVA tests; ^#^ *p* < 0.05, ^##^ *p* < 0.01, with unpaired t-test with Welch’s correction (9 m mdx vs. 9 m mdx + MS-444)

 Following the effects of HuR inhibition on fibroblasts in vitro behaviour, we treated DCM-affected 9 m mdx mice (*n* = 6 per group) with intraperitoneal IP injections of MS-444 (10 mg/kg body weight), or vehicle control 2 times/week for 40 days. Creatine kinase (CK) in the myocardium plays a crucial role in ATP production, ensuring the energy supply necessary for maintaining normal contractile function; disruptions in cardiac energy metabolism are critically involved in pathological hypertrophy and adverse cardiac remodelling, potentially linked to mitochondrial dysfunction and redox imbalance [51]. Building on previous studies demonstrating the downregulation of CK in a doxorubicin-treated mouse model of breast cancer, which showed drug-induced attenuation of cardiac fibrosis [52], we investigated circulating CK levels in 9m mdx mice treated with MS-444 and revealed a significant CK downregulation in these mice (Supplementary Fig. 3 A). In addition, considering recent studies suggesting a potential link between liver injury and cardiac pathologies [53, 54], we assessed liver function markers and found that both aspartate aminotransferase (AST) and alanine aminotransferase (ALT) levels, as well as the AST/ALT ratio, were significantly reduced following MS-444 treatment (Supplementary Fig. 3B). To further investigate the effects of MS-444 on the development of DCM in mdx mice, we conducted transthoracic echocardiography on both MS-444- and vehicle-treated 9-month-old mdx mice (Fig. 2).

MS-444-treated mdx mice showed significant smaller hearts than the untreated counterparts (Fig. 2A), as evidenced by reduced left ventricular weight (LVW) and a trend towards reduced LVW normalized to body mass weight (LVW/BW), which suggested decreased hypertrophy (Fig. 2B). The thickness of the intraventricular septum (IVS) during diastole (IVSD) and systole (IVSS), as well as the thickness of the left ventricular posterior wall (LPW) during diastole (LPWD) and systole (LPWS), normalized to body weight, showed no significant differences between groups. However, IVSD was significantly increased in dystrophic mice compared to age-matched C57Bl controls (Table 2). Conversely, LV volumes in diastole (LV vol D) and systole (LV vol S) were significantly decreased in mdx mice following HuR inhibition and similar trends were observed for Left Ventricle Internal Diameter at diastole (LVIDD) and systole (LVIDS), indicating amelioration of cardiac dilation in treated mice compared to controls (Fig. 2C). Systolic dysfunction was unaffected in both untreated and MS-444-treated mdx mice compared to wild type controls, as shown by preserved fractional shortening (FS) and ejection fraction (EF) (Table 2). Instead, mdx mice showed a significantly shorter isovolumic relaxation time (IVRT) than C57Bl, which could be indicative of elevated LV filling pressure secondary to mitral valve stenosis, while treatment with MS-444 partially restored physiological values (Fig. 2C). No significant effect of the treatment on the function of the mitral valve (MV) was observed (Table 2), while MS-444 tended to restore the function of the right ventricle (RV) of mdx mice to wild-type levels as shown by a rescue of RV outflow tract (RVOT) and RVOT-Velocity Time Integral (RVOT-VTI) in treated mdx mice (Fig. 2D).

**Table 2:**
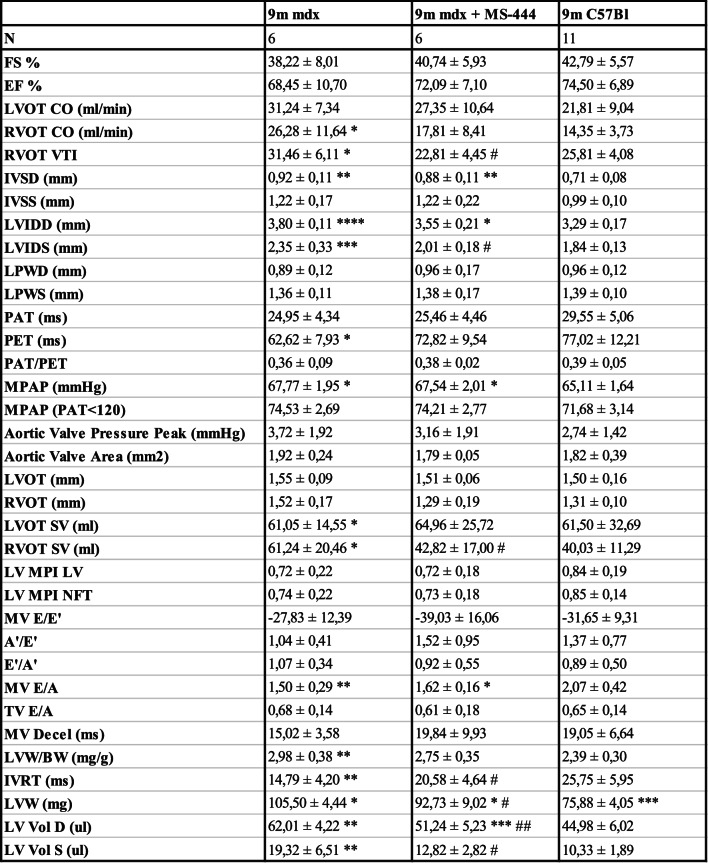
Ecocardiographic parameters in Mdx vehicle, Mdx + MS-444 and C57Bl mice

Mean ± SD. mdx vs. mdx + MS-444: *p < 0.05, **p < 0.01, ***p < 0.001, ****p < 0.0001 with unpaired t test with Welch’s correction. mdx, mdx + MS-444 and C57Bl: ^#^ *p* < 0.05, ^##^ *p* < 0.01, ordinary one-way ANOVA, Tuckey multiple comparison test

### Fibrotic development and Ki-67/HuR expression are modulated by MS-444 treatment

**Fig. 3:**
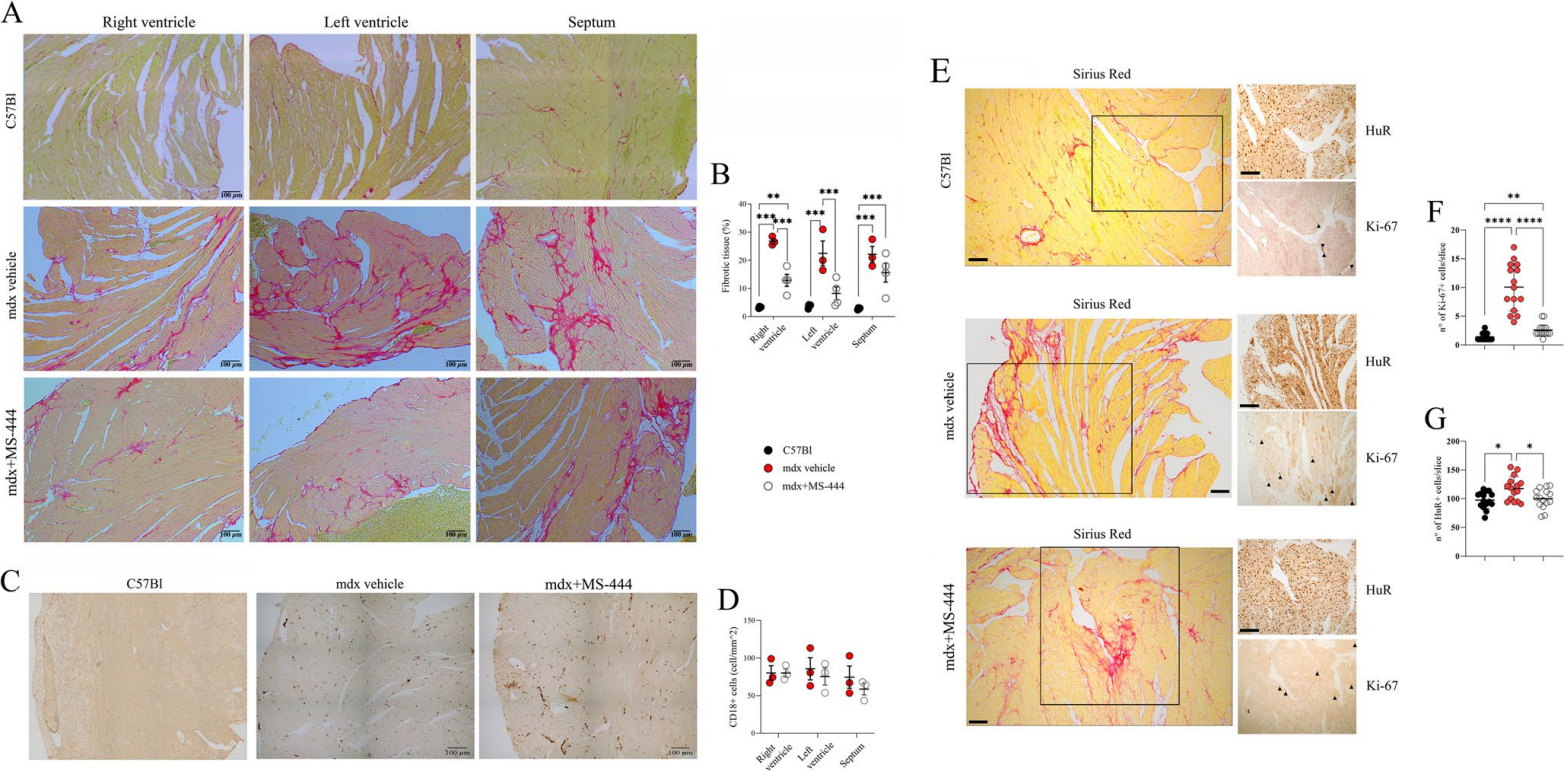
Fibrotic development in mdx cardiac tissues following MS-444 treatment. Representative images of cardiac tissues of 9 m wild type mice, mdx mice treated with MS-444 and vehicle control (*n* = 4 each) following the PicroSirus Red staining (**A**) and the relative quantification of the percentage of fibrotic tissue (**B**). Representative images of cardiac tissues of 9 m C57Bl, mdx and 9 m mdx + MS-444 (*n* = 4 each) showing the CD18 + cells (**C**) and the relative histogram representing the amount of Ki-67 per slice (**D**). Representative images of cardiac tissues of 9 m C57Bl, mdx and 9 m mdx + MS-444 (*n* = 4 each) showing the Sirius Red staining and – in higher magnification – the HuR + cells and the Ki-67 + cells (identified by black arrowheads) (**E**). Histogram representing the number of cells expressing Ki-67 (**F**) and HuR (**G**) per slice in 9 m wild type mice, mdx mice treated with MS-444 and vehicle control (*n* = 4 mice; *n* = 4 images per mice). All the images were taken at 20X. Scale bar: 100 μm. Data information: *p < 0.05, **p < 0.01, ***p < 0.001, ****p < 0.0001 with Brown-Forsythe and Welch ANOVA tests

Consistent with these findings, we observed a significant reduction in fibrosis in both the right and left ventricles of MS-444-treated mdx mice following PicroSirus Red staining. However, no modulation of the septum was noted (Fig. 3A and B). Conversely, the percentage of inflammatory cells expressing CD18 marker in cardiac tissues of mdx mice was not affected by MS-444 treatment (Fig. 3C and D).

Taking into accounts in vitro experiments, we evaluated the behavior of cardiac fibroblasts and the expression of HuR in mdx tissues. Although the adult heart is largely postmitotic, a population of proliferating cardiac progenitor cells has been identified in damaged tissue [55] and fibroblast and myofibroblast proliferation plays a central role in the development of cardiac fibrosis [56]. In line with the anti-fibrotic effects of MS-444 in mdx mice, we observed a reduction in the proliferative capacity of cells in the ECM regions, as indicated by Ki-67 staining [57]. We quantified Ki-67 + nuclei per animal and found a significant decrease in the number of these cells in MS-444-treated mdx mice compared to age-matched mdx controls, although their numbers remained higher than those in C57Bl controls (Fig. 3E and F). Notably, Sirius Red staining revealed that many Ki-67 + cells were located in fibrotic areas, further supporting the role of HuR inhibition in the modulation of pro-fibrotic cell development (Fig. 3E and F). Similarly, we counted HuR-expressing cells and observed a trend that mirrored the Ki-67 staining results (Fig. 3E and G).

### Transcriptomic and proteomic features of MS-444-treated Mdx mice

**Fig. 4:**
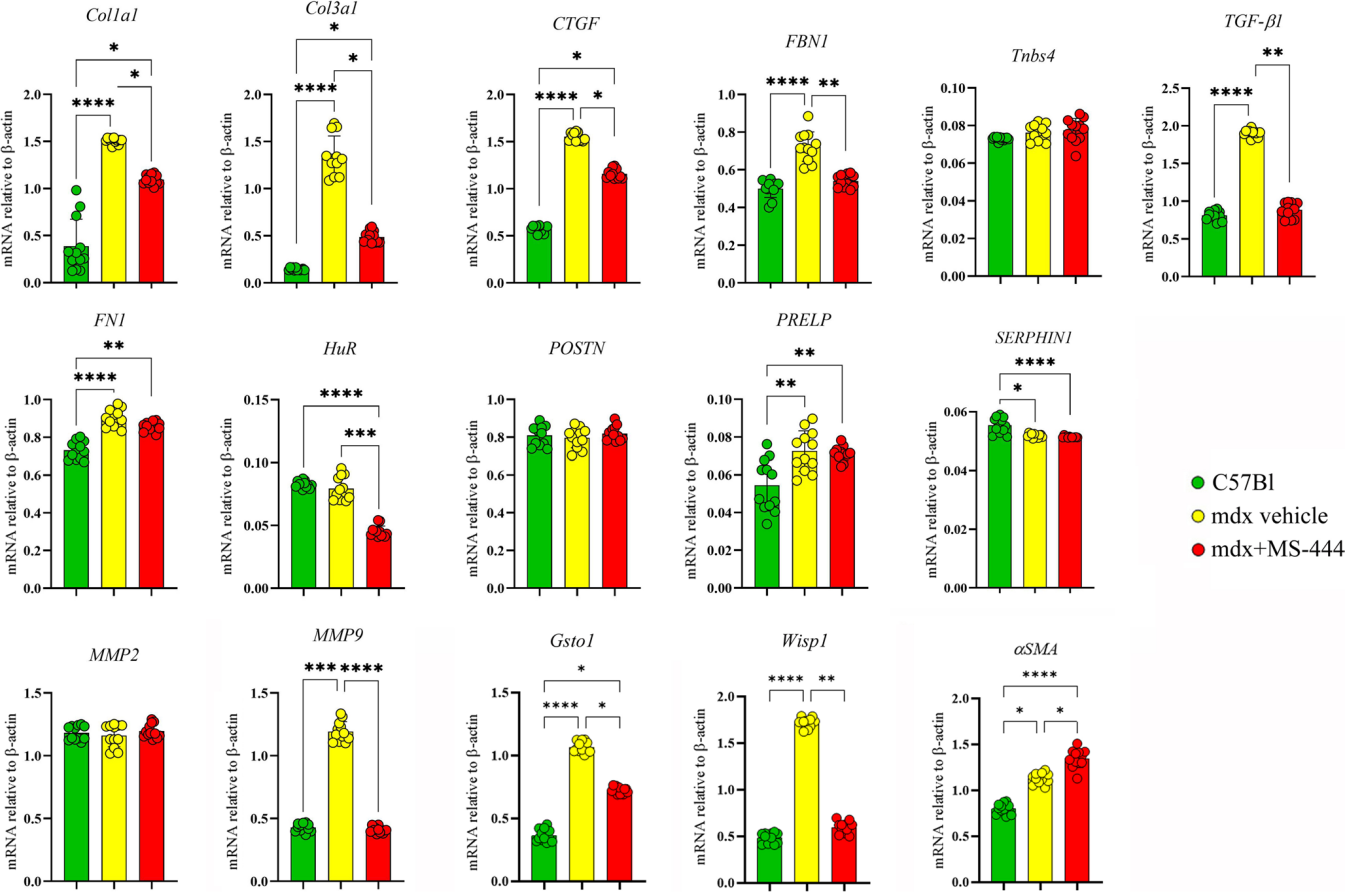
MS-444 affects pro-fibrotic genes’ expression in mdx cardiac tissues. Digital PCR evaluation of genes commonly associated to cardiac dysfunctions in mdx cardiac tissues. Data were obtained from 3 independent experiments with the 3 animals/group each experiment. Abbreviations: collagen isoforms 3a1 and 1a1 (*Col3a1* and *Col1a1*); connective tissue growth factor *(CCN2/CTGF);* fibrillin-1 *(FBN1);* Proline And Arginine Rich End Leucine Rich Repeat Protein *(PRELP);* Matrix Metallopeptidase 2 and 9 *(MMP2 and MMP9);* Thrombospondin-4 *(thbs4);* periostin *(POSTN);* fibronectin *(FN1); *WNT1-inducible signaling pathway protein 1* (WISP1/ccn4); *Glutathione S-Transferase Omega 1* (Gsto1).* Data are presented as mean ± SD. **p < 0.01; ***p < 0.001; ****p < 0.0001 one-way ANOVA Kruskal-Wallis test

**Fig. 5:**
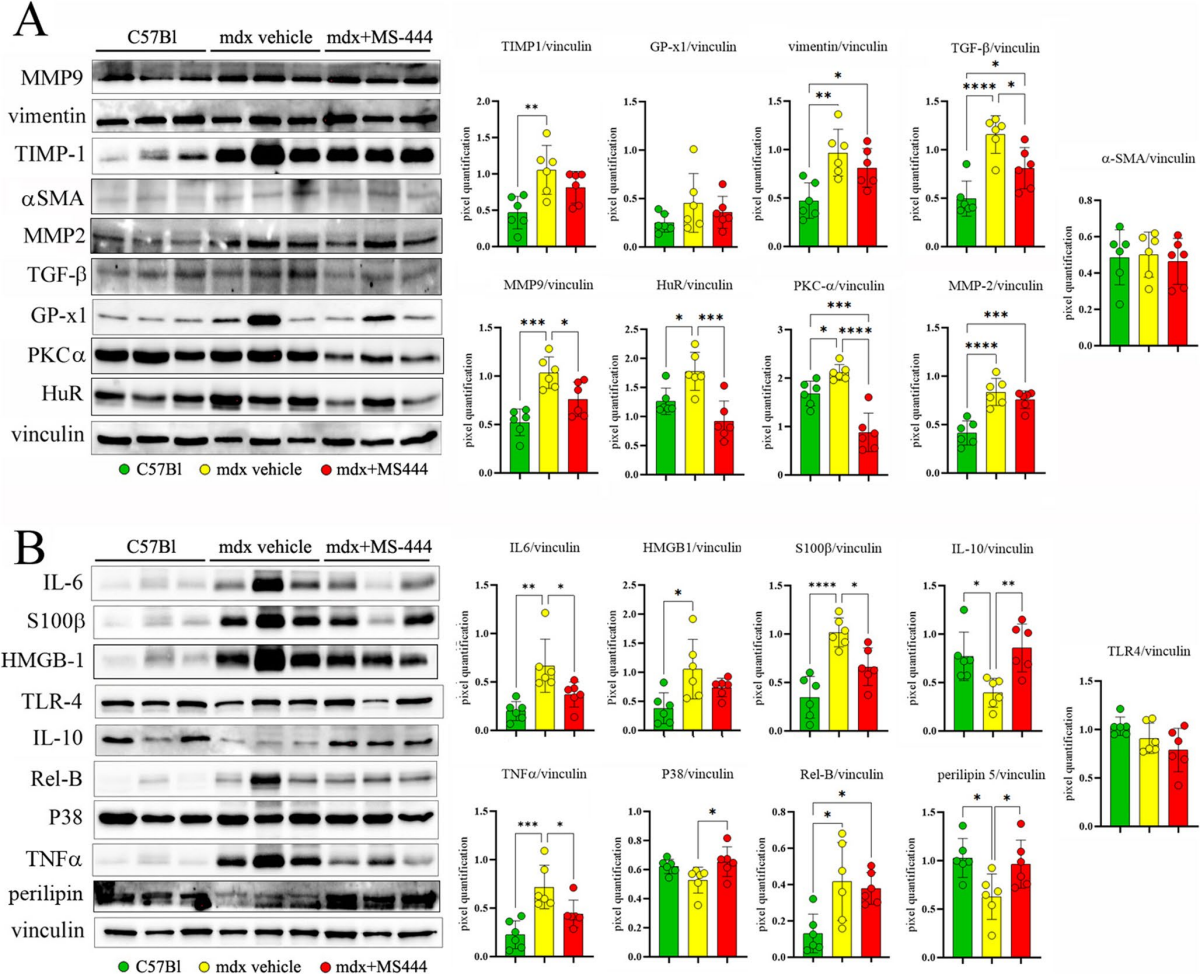
Proteomic evaluation of fibrotic and inflammatory mediators in mdx cardiac tissues following MS-444 treatment. Cropped images of representative WB analysis of cardiac muscle of 9 m mdx mice treated with MS-444 and vehicle control and age-matched C57Bl ones (*n* = 3 each, two independent experiments) showing the expression of cardiac fibrotic enhancers (**A**), alarmins and other proteins involved in anti-/pro-inflammatory signalling and fatty acid metabolism (**B**). Data information: densitometric data were normalized on vinculin and expressed as mean ± SD (*p < 0.05, **p < 0.01, ***p < 0.001, ****p < 0.0001 ordinary one-way ANOVA, Tuckey multiple comparison test)

Hypertrophic stimuli affecting cardiac myocytes determine the secretion of TGF-β that in turns activates the myofibroblasts and the remodeling of ECM [45], leading to cardiac fibrosis [58, 59]. As depicted in different works [24, 60], we showed that MS-444 treatment significantly down-regulated the expression of *TGF-β* in cardiac tissues, resembling the C57Bl one (Fig. 4). In agreement with previous findings on cardiac fibroblasts [22], the inhibition of HuR determined a down-regulation of collagen isoforms (*Col3a1* and *Col1a1*) and *connective tissue growth factor (CCN2/CTGF)* in MS-444-treated mdx mice, thus maintaining a partial over-expression related to C57Bl animals (Fig. 4). According to its role in processing ECM substrates [56], we found that *matrix metalloproteinase 9 (MMP9)* was diminished in treated mdx mice, as well as *fibrillin-1 (FBN1). Proline And Arginine Rich End Leucine Rich Repeat Protein (PRELP)* is normally upregulated in inflamed and fibrotic hearts. Accordingly, we found a dramatic over-expression of *PRELP* in mdx mice and a partial over-expression in MS-444-treated mice compared to C57BL ones (Fig. 4). *Serphin 1*, also known as *Plasminogen activator inhibitor-1 (PAI-1)* protects against cardiac fibrosis by inhibiting urokinase-type plasminogen activator (uPA) through TGF-β signaling or by regulating cardiomyocyte-derived fibrogenic signals [61]. Consistent with previous findings [62], we found that *Serphin-1* was down-regulated in dystrophic mice related to C57Bl animals (Fig. 4). Next, we analyzed other genes previously associated to HuR in a pro-inflammatory background [24] to assess whether inflammation and myofibroblast presence were fundamental to determine fibrotic development in our experimental conditions. We found a significant reduction in *WNT1-inducible signaling pathway protein 1 (WISP1/ccn4)* and *Glutathione S-Transferase Omega 1 (Gsto1)* after treatment with MS-444, indicating a strong effect of the compound in reducing both inflammation and fibrosis-related pathways. *αSMA* showed significant differences between mdx and mdx + MS-444 groups and - most importantly - among dystrophic mice and control ones (Fig. 4): transcriptomic data were not confirmed by WB (Fig. 5A). This suggests that MS-444 effectively modulates specific markers associated with inflammation and fibrosis (*Gsto1* and *WISP1*), while αSMA - likely a more specific marker of activated myofibroblasts - may be less sensitive to the treatment or involved in mechanisms not directly targeted by MS-444, and it could also suggest that MS-444 does not alter the differentiation of fibroblasts into myofibroblasts.

Subsequently, we explored whether the modulation of HuR influenced these pathogenic mediators from a proteomic point-of-view. In accordance with data coming from transcriptomic analysis, we demonstrated a significant down-regulation of HuR, MMP-9 and TGF-β following MS-444 treatment (Fig. 5A). While we did not observe a significant difference in α-SMA levels between groups, we found that MMP-2 was upregulated in dystrophic cardiac tissues compared to C57Bl controls. Importantly, this elevation of MMP-2 was diminished following MS-444 treatment. MMP-2 plays a critical role in regulating ECM degradation and turnover, as well as modulating myofibroblast activity [63]. Additionally, MMP-2 interacts with myosin light chain and troponin I, influencing cardiomyocyte function and facilitating recruitment of inflammatory cells such as macrophages, thereby exacerbating the cardiac phenotype in murine models [64] (Fig. 5A).

Our analysis revealed that HuR inhibition led to a reduction in the expression of Tissue Inhibitor of Metalloproteinases 1 (TIMP1) while GP-x1 modulation was not significant. TIMP-1 - together with TIMP-2 - is correlated to the development of cardiac interstitial fibrosis [65], possibly mediating CD63-Integrin β1 interaction [66]. Notably, we observed that vimentin – a key marker of cardiac fibroblasts involved in cellular mechanoprotection and ECM regulation [67] – was slightly downregulated in treated mdx mice rather than mdx related to control mice (Fig. 5A). Furthermore, given the suggestion that PKC-α modulates cardiac fibroblast proliferation [68] and tissue fibrosis through its effects on *galectin-3* expression [69], we found that MS-444 significantly reduced PKC-α expression in mdx mice (Fig. 5A).

As HuR allows the expression of HMGB-1 by acting on miR-1192 in the first wave of myogenesis [70], we explored the expression of the alarmins and other inflammatory mediators, such as IL-6 and TNF-α in cardiac tissues. Accordingly, MS-444 decreased the cardiac expression of HMGB1 and significantly of S100 calcium-binding protein B (S100-β) in mdx mice (Fig. 5B). Furthermore, we observed a rescue of Perilipin-5 expression in MS-444-treated mdx mice, which plays a crucial role in regulating intracellular lipid droplets (LDs) and coordinating fatty acid metabolism. Conversely, the level of IL-10 increased in MS-444 treated mice, reaching levels comparable to healthy C57Bl mice (Fig. 5B).

**Fig. 6:**
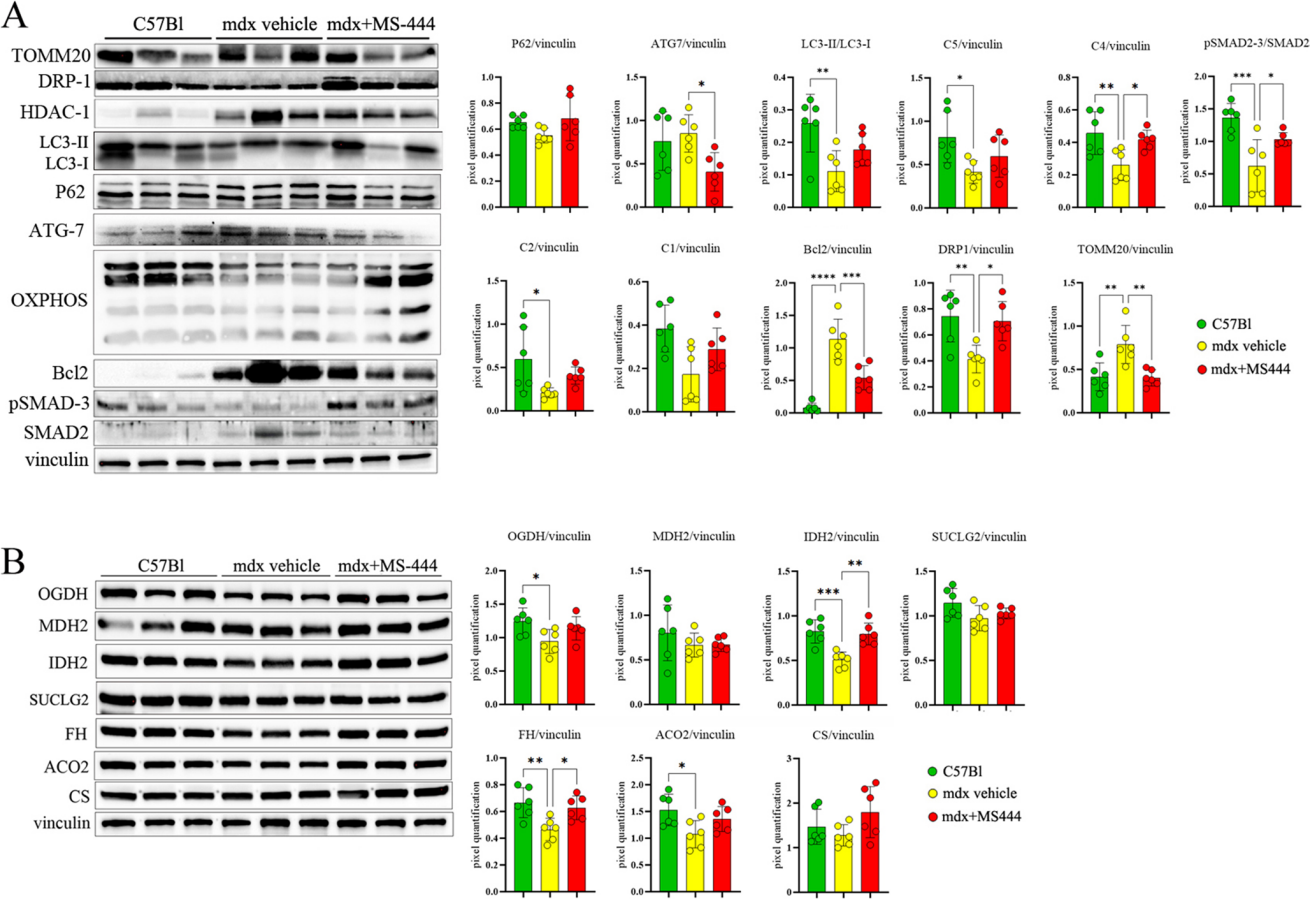
Evaluation of proteins involved in mitochondrial functions, apoptosis and TCA cycle in mdx cardiac tissues following MS-444 treatment. Cropped images of representative WB analysis of cardiac muscle of 9 m mdx mice treated with MS-444 and vehicle control and age-matched C57Bl ones (*n* = 3 each, two independent experiments) showing the expression of proteins involved in (**A**) mitochondrial functions, oxidative phosphorylation and apoptosis/mitophagy and (**B**) TCA cycle. Data information: densitometric data were normalized on vinculin and expressed as mean ± SD (*p < 0.05, **p < 0.01, ***p < 0.001, ****p < 0.0001 ordinary one-way ANOVA, Tuckey multiple comparison test)

Drawing insights from an established cardiovascular disease model by Liu et al. [71], which demonstrated that HuR inhibition affects autophagy and subsequently apoptosis, we observed an augmentation of p62 and the restoration of C4-MTCO1 expression, while B-cell lymphoma 2 (BCL-2) decreased. The down-regulation of BCL-2 was in line with previous results suggesting an upregulation in fibrotic hearts and especially in tissues affected by DCM [72]. Furthermore, the expression of dynamin-related protein 1 (DRP1), involved in mitochondrial organization, was rescued in treated mdx mice, and the protein level of translocase of the outer mitochondrial membrane 20 (TOMM20), crucial for protein targeting to the mitochondrion, was restored to normal levels (Fig. 6A).

The tricarboxylic acid cycle (TCA) cycle plays a pivotal role in maintaining the energy requirements and regulating the contractile functions of cardiomyocytes, while also controlling myocardial oxidative stress. Dysregulation of these processes is implicated in the pathogenesis of DCM [73, 74]. In accordance, Adamoski et al. demonstrated that HuR is actively involved in modulating glutamine mRNA in cancer cells, a process crucial for ATP synthesis through the TCA cycle and for redox balance regulation [75]. Notably, our investigation revealed the up-regulation of Oxoglutarate Dehydrogenase (OGDH), Fumarate Hydratase (FH), Aconitase 2 (ACO2), and Isocitrate Dehydrogenase (NADP(+)) 2 (IDH2) in mdx mice treated with MS-444, aligning their expression levels with those observed in C57Bl tissues (Fig. 6B). In contrast, we did not reveal any significant difference in protein expression of Malate Dehydrogenase 2 (MDH2), Succinate-CoA Ligase GDP-Forming Subunit Beta (SUCLG2) and citrate synthase (CS) (Fig. 6B).

## Discussion

In DMD patients as well as in murine animal models, the absence of dystrophin and the inflammatory background determine the development of DCM, that is not dependent on muscular pathology [76] or modulation of vasculature [77]. More in general, the development of DCM in dystrophin-deficient tissues is caused by the remodeling of ECM that leads to dysfunctions in the collagen fibrils that transmit the contractile force and alterations in other ECM proteins (as fibronectin and glycoproteins) and in cell-matrix interactions: all together, these pathological cues cause the rising of fibrosis and the impairment of cardiac functionality.

Since many patients are affected by DCM, it represents the most common cause of mortality: unfortunately, several therapies were blunted by limits in target selectivity and cardiotoxicity using modulators of myofibroblasts or microenvironmental signaling or RNA-dependent pro-fibrotic pathways [12–14]. Different studies on myocardial infarction clearly demonstrated that RBPs had to be considered as potential therapeutic target for cardiac pathologies as they regulated cellular hypoxia, oxidative stress, pro-inflammatory responses, and fibrotic repair [78]. In line, the inhibition of HuR in TGFb1–treated cardiac fibroblasts suppressed myofibroblast differentiation and proliferation and blocked the development of fibrosis, possibly affecting the expression of cyclins D1 and A2 [21]. HuR is fundamental in the regulation of several cell types (hematopoietic progenitor cell, T cell, B cell antibody response) [79] and it is involved in cardiomyocyte hypertrophy [20], cardiac fibrosis development [22] and myofibroblasts proliferation [24]. Pharmacokinetic (PK) and pharmacodynamic (PD) data on MS-444 remain limited; however, available evidence indicates that it effectively modulates HuR activity at low micromolar concentrations with tolerable toxicity in animal models [33, 40].

The data presented in this study investigate the potential links between DCM associated with DMD and the modulation of the RNA-binding protein HuR. The analysis involved examining cardiac tissues from mdx mice at different stages of disease progression, ranging from pre-onset to advanced DCM, and comparing them to age-matched healthy C57Bl mice. Initially, at the pre-onset stage, there were no significant differences observed in HuR expression between mdx mice and healthy controls. However, as the mice aged and DCM progressed, HuR expression notably increased in mdx mice compared to the control group. We specifically observed the highest level of HuR overexpression in 18-month-old mice. Cardiac fibroblasts play pivotal roles in ECM remodeling, and fibroblasts senescence is implicated in the development of cardiac fibrosis [80]. Innovative work from Tanter lab demonstrated that HuR inhibition through KH-3 in murine cardiac fibroblasts blunted the expression of pro-inflammatory genes and other ECM-related proteins, as *collagen* and *periostin* [24].

Following isolation of cardiac fibroblasts from mdx and C57Bl mice, we observed that MS-444 treatment diminished their proliferation rate and reduced their mRNA HuR expression. Furthermore, we found a clear accumulation of HuR protein in the nuclear fraction with a corresponding reduction in the cytoplasm upon MS-444 treatment. These data are consistent with reports that MS-444 primarily inhibits HuR function by blocking its nucleocytoplasmic shuttling [81, 82], suggesting that the resulting nuclear retention of HuR not only reduces its cytoplasmic RNA-binding activity but also leads to autoregulatory downregulation of HuR mRNA and protein levels [83]. Moreover, the observed elevation of HuR protein levels during DCM progression, despite stable HuR mRNA levels, likely reflects an increase in HuR translational efficiency rather than changes in transcription. This is consistent with known post-transcriptional regulatory mechanisms of HuR, where autoregulatory feedback loops maintain HuR homeostasis through modulation of mRNA stability and alternative polyadenylation rather than direct transcriptional repression. Thus, MS-444 primarily acts at the post-transcriptional and post-translational levels by interfering with HuR’s RNA-binding capacity and subcellular localization, thereby diminishing its autoregulatory expression feedback.

In accordance with a potential therapeutic effect of targeting HuR in mitigating inflammation associated with DCM, pro-fibrotic and -inflammatory cytokines IL6, TLR4, and TNF-α were downregulated in MS-444-treated fibroblasts from mdx hearts compared to vehicles. Similarly, MS-444 inhibited the HMGB1-p21 axis of senescence in mdx cardiac fibroblasts, thus representing a mechanistic pathway responsive to MS-444, that potentially mitigates DCM features by reducing fibrotic signals and preventing detrimental myocardial remodeling [84].

Since it is well accepted that fibroblast-to-myofibroblast differentiation and alterations in ECM structural properties are fundamental steps in the initiation of fibrosis [85], we suggest that the down-regulation of fibronectin and collagen I in mdx MS444-treated cells, as well as Cyclin D1, could represent different steps of the involvement of HuR in controlling the fate of cardiac myofibroblasts and consequently fibrosis.

Following these promising in vitro evidences, we then investigated the impact of inhibiting HuR in 9-month-old mdx mice with DCM using MS-444. Firstly, considering the role of CK reaction in myocardial ATP production and its importance in cardiac energy metabolism, the observed down-regulation in treated mice suggests a potential restoration of normal cardiac function following HuR inhibition. Furthermore, recognizing the potential link between liver injury and cardiac pathologies, we assessed the effect of the treatment on liver markers. Using MS-444, we found a significant decrease in AST/ALT markers post-treatment indicating a potential attenuation of liver-related complications associated with cardiac dysfunction. In terms of cardiac function, HuR inhibition was effective in counteracting multiple features of DMD-related DCM, like hypertrophy, LV dilation, and RV dysfunction. MS-444-treated mdx mice exhibited significantly reduced LV hypertrophy as indicated by lower LVW and LVW/BW than untreated mdx animals. Additionally, reductions in LV vol D/S following HuR inhibition were indicative of reduced dilation and improved LV function. Finally, the rescue of RVOT and RVOT-VTI in MS-444-treated mdx mice indicated a similar improvement of the RV functionality. Overall, these findings suggest that HuR inhibition holds promise as a therapeutic strategy for improving cardiac function in DCM-affected mdx mice.

Importantly, the transcriptomic characterization of cardiac tissues in mdx treated with MS-444 mice revealed the downregulation of pro-fibrotic genes as *TGF-β*, *CTGF*,* FBN1* and *collagen* genes. *FBN1* is the most important component of the microfibrils that regulates the elasticity and the stiffness of the ECM and *FBN1* over-expression determines the weakening and the alteration of immune response [86]. *CCN2* is normally expressed in the ECM of injured heart and it is correlated to maladaptive fibrotic remodeling [87]: indeed, it is regulated by TGF-β [88].

In line, the proteomic experiments suggested that MS-444 determined the down-regulation of pro-inflammatory cytokines, as TNF-α and IL-6 and of important fibrotic mediators, as vimentin, TGF-β, and MMP-9 whose functional regulation of ECM components (collagen, fibronectin, laminin) is necessary to limit these pathological features [33]. Moreover, we found a modulation of PKC-α in MS-444-treated mice, whose involvement in cardiac fibrosis is mediated by *galectin-3* and affects directly *collagen 1a1/fibronectin* [89] and *collagen 3a1/α-SMA* [68] expression, that are commonly altered in dystrophic tissues. The dysfunction of oxidative respiratory chain and metabolic-related enzymes gained importance in the last years as fundamental players in the development of cardiac fibrosis. Reduced OXPHOS activity in myocardium dramatically affected the mitochondrial function, causing the rising of oxidative stress and inflammatory signaling [90]. As we observed partial restoration of OXPHOS proteins and TOMM20 to normal levels and we noted a rescue of DRP1 expression in MS-444-treated mdx mice, we suggest a potential mitigation of DCM mitochondrial dysfunction following MS-444 treatment. Importantly, mitochondria activities are strictly associated to cardiac sodium channel [91, 92] - whose regulatory network is mediated by HuR [29] – and more importantly are fundamental to directly regulate bioenergetic machinery, largely involved in cardiac fibrosis and pathological remodeling [93], or indirectly to limit ROS and their detrimental functions on ECM functions [94].

Accordingly, we found modulation of Perilipin-5 driven by MS-444: its absence in murine cardiomyocytes can lead to impaired glucose utilization, subsequently contributing to the development of cardiac hypertrophy [95]. Additionally, alterations in fatty acid β-oxidation and lipotoxicity can compromise the antioxidative capacity of cardiomyocytes, leading to the up-regulation of ROS, oxidative stress, and impairment of mitochondrial functions [96].

The secretion of alarmins from necrotic cardiomyocytes determines the expression of pathological cytokines and the recruitment of inflammatory cells [97]. MS-444 treatment decreased cardiac expression of HMGB1, S100-β and Perilipin-5 in mdx mice, indicating a potential therapeutic effect on inflammation and metabolic dysregulation associated with oxidative stress.

It was demonstrated that HuR modulates the translation efficiency of mRNAs encoding key metabolic mediators, as glucose transporters and glycolytic proteins [98]: taken together with the observed down-regulation of TCA enzymes in cardiac tissues of mdx mice treated with MS-444, our treatment potentially restores cardiac metabolic homeostasis, as well as cellular energy production, whose alteration determine cardiac fibroblasts’ activation and fibrosis’ spreading [99].

Our data are similar to other experimental evidence recently obtained by others, following the inhibition of HuR in animal model of myocardial ischemia/reperfusion (I/R) injury. These studies noted a significant reduction of tissue pathological remodeling, possibly due to a decrease in pro-inflammatory proteins and diminished recruitment/activation of pro-fibrotic monocytes/macrophages [100].

As previously described [101], various metabolic phenomena, including glycolysis, amino acid utilization, and decreased oxidative phosphorylation in immune cells [102] as well as the over-expression of lactate, succinate, HIF1-α, and TCA cycle intermediates [103], are influenced by metabolic dysfunctions. In mdx mice, the altered metabolic reprogramming of cardiac cells may impair their coordination with macrophages and lymphocytes, affecting pathways that are essential not only for providing the energy required for heart function but also for maintaining the structural and spatiotemporal homeostasis of cardiac tissue. This metabolic dysfunction leads to the activation of myofibroblasts and exacerbates cardiac fibrogenesis.

All these data align with the significant reduction of fibrosis observed in both the right and left ventricles of MS-444-treated mdx mice but, at the same time, are not limited only to this pathway, as its effects on apoptosis, inflammation and metabolism are evident. Intriguingly, since all these signalling cascades are involved in fibrosis - as in the initial phase or in the propagation -, the multi-step effects of MS-444 could retain significant ameliorative effects on DCM in mdx mice. Moreover, considering that MS-444 is successfully used to inhibit HuR activity in cancer cells [33, 40, 104, 105] as well as in cystic fibrosis [39] and immune pathologies [106] and clinical trials involving HuR inhibitors are already underway [30], our preclinical results provide a strong rationale for further investigations to determine the true therapeutic benefit of HuR inhibition in mitigating cardiac dysfunctions in DMD patients. MS-444 was originally characterized as an MLCK inhibitor given the critical role of this protein in cardiac muscle contraction and cellular motility, potential off-target effects on MLCK in cardiac and mdx models cannot be excluded. However, studies in colorectal and glioblastoma cancer cells have shown that MS-444 does not significantly affect MLCK expression or downstream MAPK signaling at effective concentrations for HuR inhibition [33, 40]. Nonetheless, the impact of MLCK inhibition by MS-444 in cardiac contexts, especially related to mdx mouse models, warrants cautious interpretation and further investigation, although the beneficial effects observed in cardiac and mdx contexts are primarily mediated via HuR inhibition. In summary, MS-444 is a widely accepted chemical tool to inhibit HuR function by impairing its RNA-binding and cytoplasmic localization, with demonstrated efficacy in diverse biological systems: collaborative efforts between basic researchers and clinicians will be essential in translating these preclinical findings into effective therapeutic interventions for improving cardiac outcomes in dystrophic patients.

## Supplementary Information

Below is the link to the electronic supplementary material.

Supplementary Material 1

## Data Availability

Source data for all main figures and extended data figures are supplied with this paper. All necessary data to evaluate the paper’s conclusions are available in the paper and Supporting Information. Other experimental data supporting the plots within this paper and other findings of this study are available from the corresponding author upon reasonable request.

## Abbreviations

MDs: Muscular Dystrophies
DMD: Duchenne Muscular Dystrophy
DM: Myotonic Dystrophy
FSHD: Facioscapulohumeral Muscular Dystrophy
DAMPs: Danger Associated Molecular Patterns
DCM: Dilated Cardiomyopathy
DGC: Dystrophin-Glycoprotein Complex
ECM: Extracellular Matrix
NO: Nitric Oxide
ACE: Angiotensin-Converting Enzyme
RBPs: RNA Binding Proteins
PUM2: Pumilio RNA Binding Family Member 2
QKI: Quaking
HuR: ELAV Like RNA Binding Protein 1
LV: Left Ventricular
Wisp1/Ccn4: WNT1-Inducible Signaling Pathway Protein 1
CTGF: Connective Tissue Growth Factor
CCN: Nephroblastoma Overexpressed
SFRP2: Safe-Secreted Frizzled Related Protein 2
SCN5A: Sodium Channel A-Subunit
MEF2C: Myocyte Enhancer Factor-2 C
PD-L1: Programmed Death-Ligand 1
IP: Intraperitoneal
FBS: Fetal Bovine Serum
MTT: Thiazol Blue Tetrazolium Bromide
BSA: Bovine Serum Albumin
PBS: Phosphate-Buffered Saline
HS: Horse Serum
Ck: Creatine Kinase
AST: Aspartate Aminotransferase
ALT: Alanine Aminotransferase
LVW: Left Ventricular Weight
LVW/BW: LVW Normalized To Body Mass Weight
IVSD: Intraventricular Septum (IVS) During Diastole
IVSS: Intraventricular Septum (IVS) During Systole
LPWD: Left Ventricular Posterior Wall (LPW) During Diastole
LPWS: Left Ventricular Posterior Wall (LPW) During Systole
LV Vol D: LV Volumes In Diastole
LV Vol S: LV Volumes In Systole
LVIDD: Left Ventricle Internal Diameter At Diastole
LVIDS: Left Ventricle Internal Diameter At Systole
FS: Fractional Shortening
EF: Ejection Fraction
IVRT: Isovolumic Relaxation Time
MV: Mitral Valve
RV: Right Ventricle
RVOT: RV Outflow Tract
RVOT-VTI: RVOT-Velocity Time Integral
I/R: Ischemia/Reperfusion
CCN2/CTGF: Connective Tissue Growth Factor
MMP9: Matrix Metalloproteinase 9
FBN1: Fibrillin-1
PRELP: Proline And Arginine Rich End Leucine Rich Repeat Protein
PAI-1: Plasminogen Activator Inhibitor-1
UPA: Urokinase-Type Plasminogen Activator
TIMP1: Tissue Inhibitor Of Metalloproteinases 1
S100-Β: S100 Calcium-Binding Protein B
LDs: Lipid Droplets
BCL-2: B-Cell Lymphoma 2
DRP1: Dynamin-Related Protein 1
TOMM20: The Outer Mitochondrial Membrane 20
TCA: Tricarboxylic Acid Cycle
OGDH: Oxoglutarate Dehydrogenase
FH: Fumarate Hydratase
ACO2: Aconitase 2
IDH2: Isocitrate Dehydrogenase (NADP( +)) 2
MDH2: Malate Dehydrogenase 2
SUCLG2: Succinate-Coa Ligase GDP-Forming Subunit Beta
CS: Citrate Synthase
WISP1/ccn4: WNT1-inducible signaling pathway protein 1
Gsto1: Glutathione S-Transferase Omega 1
PK: Pharmacokinetic
PD: Pharmacodynamic
