# Supplementary material for: Inhibition of HuR/ELAVL-1 attenuates fibrotic progression in Mdx mice with dilated cardiomyopathy
Source: Cell Mol Life Sci. 2025 Dec 30;83(1):49. doi: 10.1007/s00018-025-05979-0 (PMC12799888; doi:10.1007/s00018-025-05979-0)

**Supplementary Figure 1**

Digital-PCR analysis of three independent experiments (n=3 each) demonstrated the overexpression of HuR in dystrophic cells related to healthy ones and – especially – in fibroblasts.

Data are presented as mean ± SD. ***p<0.001; ****p<0.0001 with unpaired t-test with Welch’s correction; and ^###^p<0.001; ^####^p<0.0001 with Brown-Forsythe and Welch ANOVA tests.


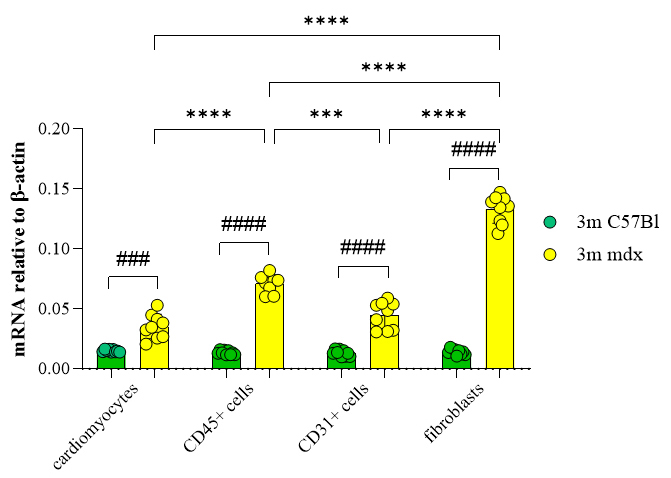


**Supplementary Figure 2**

Digital-PCR analysis of two independent experiments (n=3 each) investigated the modulation of the expression of pro-inflammatory and fibrotic genes in cardiac fibroblasts treated with different dosage of MS-444 (20 μm and 50 μm).

Data are presented as mean ± SD. **p<0.01; ***p<0.001; ****p<0.0001 with unpaired t-test with Welch’s correction; and ^###^p<0.001; ^####^p<0.0001 with ordinary one-way ANOVA test.


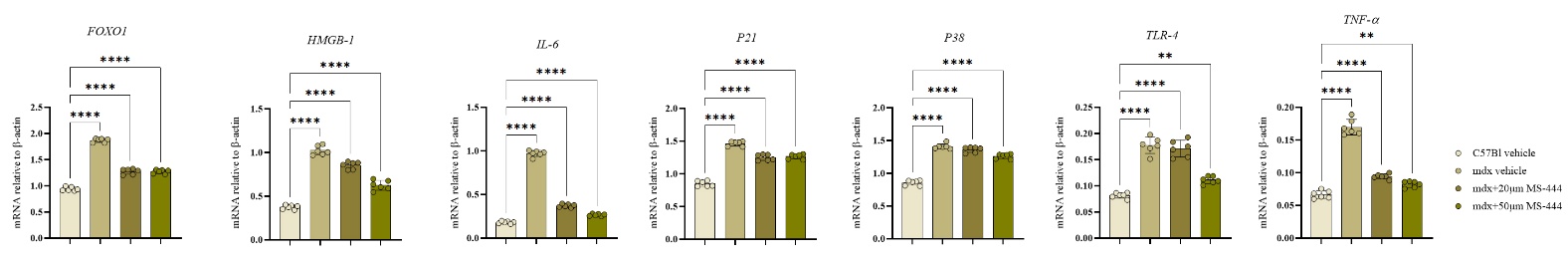


**Supplementary Figure 3**

Serum analysis of CK (A) and AST/ALT (B) in Cropped images of representative WB analysis of cardiac muscle of 9m mdx mice treated with MS-444 (n=6) and vehicle control (n=10) and age-matched C57Bl ones (n=7).

Data information: *p<0.05, **p<0.01, ***p<0.001 with ordinary one-way ANOVA, Tuckey multiple comparison test.


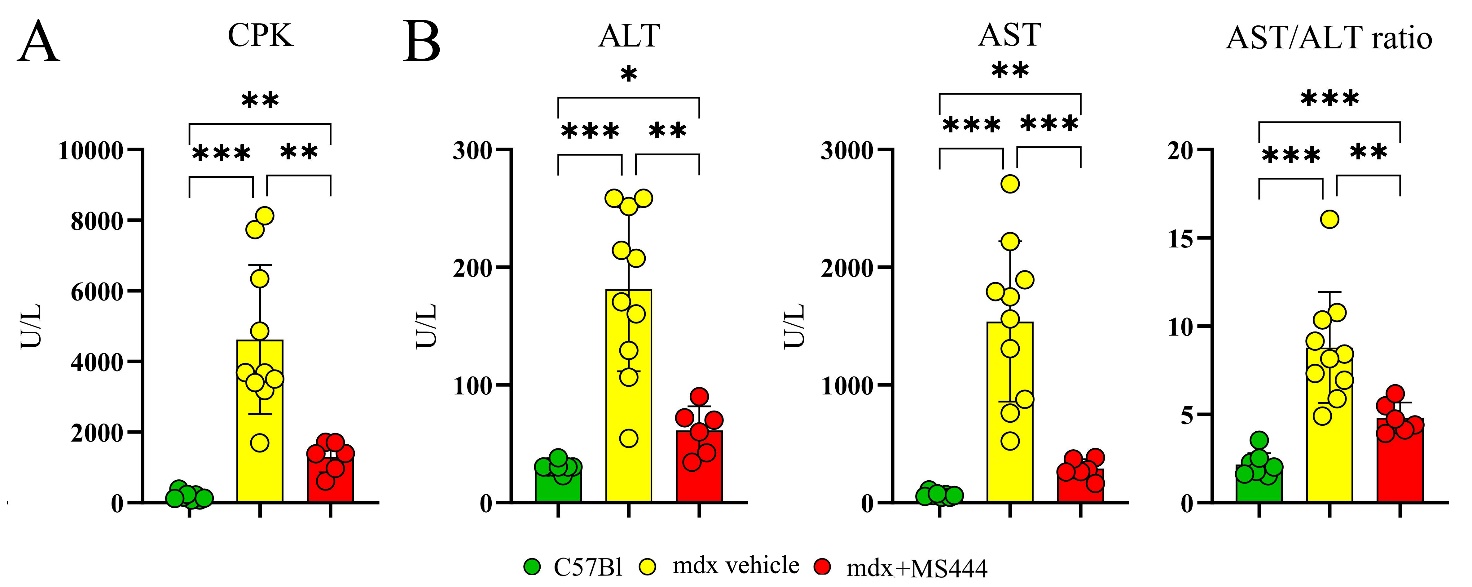

Supplement: Supplementary file 1 — Supplementary Material 1 [file 18_2025_5979_MOESM1_ESM.docx]
